# Identification of key genes related to growth of largemouth bass (*Micropterus salmoides*) based on comprehensive transcriptome analysis

**DOI:** 10.3389/fmolb.2024.1499220

**Published:** 2024-12-11

**Authors:** Dayan Hu, Jieliang Jian, Jinpeng Zhang, Xiaojun Xu, Shu Wang, Cuiping Gong, Yuanqin Zhang, Pengcan Zhu, Zhimin Gu, Wenzhi Guan

**Affiliations:** ^1^ Huzhou Key Laboratory of Innovation and Application of Agricultural Germplasm Resources, Huzhou Academy of Agricultural Sciences, Huzhou, China; ^2^ State Key Laboratory for Managing Biotic and Chemical Threats to the Quality and Safety of Agro-products, Institute of Hydrobiology, Zhejiang Academy of Agricultural Sciences, Hangzhou, China; ^3^ Huzhou Rongsheng Fishery Technology Company, Huzhou, China

**Keywords:** largemouth bass, different growth periods, histological analysis, DEGs, transcriptome

## Abstract

**Introduction:**

Largemouth bass is an economically important farmed freshwater fish species that has delicious meat, no intermuscular thorns, and rapid growth rates. However, the molecular regulatory mechanisms underlying the different growth and developmental stages of this fish have not been reported.

**Methods:**

In this study, we performed histological and transcriptomic analyses on the brain and dorsal muscles of largemouth bass at different growth periods. The brain and muscle tissue were dehydrated, embedded, sliced and stained with hematoxylin-eosin. Images were captured under a microscope and acquired using a microphotographic system. Differential expression between groups was analyzed using DESeq2. GO functional analysis and KEGG pathway analysis were then performed for differentially expressed genes. RT-qPCR validates the reliability of transcriptome sequencing data.

**Result:**

Smaller fish had more new muscle fiber numbers and wider intermuscular spaces compared to big specimens. Axons and nerve fibers were more pronounced in the telencephalons of big fish than in small fish. A total of 19,225 differentially expressed genes (DEGs) were detected in the muscle tissue, among which 7,724 were upregulated and 11,501 were downregulated, while a total of 5,373 DEGs were detected in the brain, among which 2,923 were upregulated and 2,450 were downregulated. GO and KEGG enrichment analyses indicated that nucleic acid binding, cytoskeletal motor activity, DNA binding, circadian rhythm, glycolysis/gluconeogenesis, and osteoclast differentiation were related to brain development while binding, cytoskeletal protein binding, biological processes, c-type lectin receptors, mitogen-activated protein kinase (MAPK) signaling pathways, and osteoclast differentiation were related to muscle growth. *Stat3*, *pparg*, *akt1*, *mapk3*, and *mapk1* genes were mainly involved in the growth and development of largemouth bass.

**Conclusion:**

These results provide novel perspectives for deepening our understanding of the mechanisms underlying the growth and development and performing genetic selection in largemouth bass.

## Introduction

Growth is one of the most important traits of economically important aquaculture animals (Sherzada et al., 2023). High growth performance in aquaculture species can not only provide high-quality protein for people but also bring direct economic benefits (Khan et al., 2023). Growth is a complex trait influenced by genes, environmental factors, and the interactions between genes and environmental factors (Li et al., 2017; Leips and Travis, 2013). For example, stocking density can significantly affect the weight gain rate, specific growth rate, and survival rate of *Litopenaeus vannamei* (Yuan et al., 2023), and the environmental factors temperature, pH, dissolved oxygen, and nutrition are closely related to the growth of fish (Perez-Sanchez et al., 2018; Sahoo et al., 2020; Johnston et al., 2003). Genes are also significantly correlated with fish growth and determine the growth potential of fish (Perry et al., 2005).

With the development of molecular genetics in fish, insights have been gained into the molecular regulatory mechanisms and pathways underlying growth traits, and such knowledge is of great economic significance for the exploration of genes related to growth in fish (Li Y. et al., 2024; Li et al., 2018). Moreover, an increasing number of studies have identified key functional genes and pathways associated with growth traits in aquatic animals. The GH/IGF system plays an integral role in the growth and development of fish and regulates growth and metabolic processes through a complex network of receptors and signaling pathways (Reindl, 2012; Fuentes et al., 2013). Many forms of GH, GH receptors (GHRs), and insulin-like growth factor receptors (IGFRs) have been identified (Sherzada et al., 2023; Pankova et al., 2017). IGF1 is the most studied and characterized member of the IGF family, and it promotes cell proliferation, differentiation, and protein synthesis by binding to IGFR and activating downstream signaling pathways (PI3K/Akt and mitogen-activated protein kinase (MAPK)) to promote the growth and development of tissues, such as muscle and bone (Duran et al., 2022). In addition to the GH/IGF axis, myogenic regulatory factors (MRFs) also regulate fish growth and development. MRFs consist of *myod*, *myog*, *myf5*, *myf6*, and *mrf4*, and their expression and functional regulation directly affect muscle health and function (Cao et al., 2023; Li Q. et al., 2024). Two genes *myog* and *myf6* are highly expressed in developing muscle tissue and are mainly involved in the differentiation process of myoblasts (Rajesh et al., 2019).

In recent years, transcriptome sequencing technology (RNA-seq) has been widely used in studies on aquatic animals, including investigations of growth development, immune response, organic evolution, and other related aspects (Pang et al., 2024; Zhong et al., 2024). Previous studies have reported that the HIF-1, AMPK, and glycolysis/glycolysis pathways play important roles in hypoxia adaptation in yellow catfish (*Pelteobagrus fulvidraco*). Moreover, *egln2*, *egln3*, *eng*, and *vegf* promote angiogenesis and erythropoiesis in the brain to increase oxygen transport during hypoxia (Zhao C. et al., 2024). In a study on *Mastacembelus armatus*, the genes *tlr5m*, *tlr5s*, *il1β*, *il8*, and *tnf* were found to play key roles in the immune response of *Aeromonas veronii* (Han et al., 2019). Dietary phospholipids can regulate skeletal development in larval largemouth bass by regulating key genes associated with bone formation, such as *twist2*, *daam1*, *bglap*, *pcolce b*, *scpp1*, and *scpp7* (Fang et al., 2023). However, the use of RNA-seq to study differential genes and signaling pathways in largemouth bass at different growth stages has not been reported to date.

Largemouth bass (*Micropterus salmoides*) is native to the United States and has been farmed throughout China owing to its strong adaptability, fast growth, rich nutrition, and high economic value (Zhang et al., 2022; Chen Q. K. et al., 2024). Largemouth bass has received extensive attention as a research hotspot in recent years, with studies focusing on its nutritional requirements, immune mechanisms, physiological regulation, and other aspects, such as the effects of feed additives on gut health, *Aeromonas hydrophila* effects on immunity and microbiota homeostasis, and identifying SNP locus markers related to growth (Yuan et al., 2021; Yuan et al., 2024; Li et al., 2017). However, studies on the molecular regulatory mechanisms of growth and development in largemouth bass at different growth stages have not been reported.

Therefore, this study performed RNA-seq analyses of the brain and muscle tissue of largemouth bass at different growth stages to explore the key genes and pathways underlying the associated changes. Overall, this study provides a reference for further research on the growth and development mechanisms of largemouth bass and other fish species.

## Material and methods

### Rearing experiment and sample collection

Largemouth bass used in this study were obtained from Huzhou Rongsheng Fishery Technology Co. For the experimental fish, we selected the same batch of fish that were hatched and reared under the same feeding management conditions. The initial stocking density was 5 tail fish at each 1 m^2^. During the feeding trials, the commercial feed containing more than 48% crude protein was fed at 7:00 and 17:00 daily, respectively. Water temperature ranged 25°C ± 2°C, pH 7.0 ± 0.5, and the dissolved oxygen lever remained above 5.0 mg/L.

Brain and muscle tissues of 1.5-month-old (small fish, total length 7.50 ± 0.6 cm, 4.50 ± 0.26 g), 4.5-month-old (medium fish, total length 22.46 ± 3.1 cm, 186.56 ± 13.52 g), and 7.5-month-old (big fish, total length 26.60 ± 2.3 cm, 368.92 ± 20.15 g) largemouth bass were taken for the experiment, respectively. Ten fish were randomly selected from the pond for each sampling and anesthetized. Three fish were randomly selected from the 10 anesthetized fish to be sampled for subsequent analytical experiments. The telencephalon and a small piece of the back muscle were placed in Servicebio solution for 24 h before histological analysis. Subsequently, the remaining brain and back muscles were collected separately into cryotubes, immediately immersed in liquid nitrogen, and then kept at −80°C for transcriptome analysis.

### Histological observe

The brain and muscle tissue were removed from the fixative, dehydrated in ethanol, equilibrated in xylene, embedded in paraffin, sliced to a thickness of 4 μm, and stained with hematoxylin-eosin (HE). Images were captured under a microscope (Nikon Eclipse E100) and acquired using a microphotographic system (Nikon DS-U3). The software of “ImageJ” was used to measure the gap between muscle fibers.

### RNA isolation, cDNA library construction sequencing

Total RNA was isolated from the muscle and brain tissues using TRIzol® Reagent according to the manufacturer’s instructions. RNA quality was determined using a 5300 Bioanalyzer (Agilent, Santa Clara, CA, United States) and quantified using an ND-2000 (NanoDrop Technologies). Only high-quality RNA sample (OD260/280 = 1.8–2.2, OD260/230 ≥ 2.0, RIN ≥6.5) was used to construct the sequencing library. Next, mRNA with polyA tails was enriched using Olig magnetic beads and cut into 300 bp fragments by ion interruption. RNA-seq libraries were prepared using these segments as a template to synthesize cDNA by 6-base random primers. After enrichment and generating highly qualified library fragments, all RNA-seq libraries were sequenced on a NovaSeq 6000 sequencer using an Illumina paired-end 2 × 150 bp run (6 Gb; Q30 > 80%) by Shanghai Majorbio Bio-pharm Biotechnology Co., Ltd. (Shanghai, China).

### Transcriptome data analysis

Raw paired-end reads were trimmed and quality was controlled using fastp with default parameters (https://github.com/OpenGene/fastp). RNA-sequencing data were filtered to remove low-quality reads, including reads with adaptors, reads with >10% of unknown nucleotides (N), reads with >50% low-quality (Qvalue ≤ 20) bases and reads with a sequence length less than 20 bp. These data were then compared to a reference genome (*M. salmoides*, GCF_014851395.1) by HISAT2.

Differential expression between groups was analyzed using DESeq2 (Love and Anders, 2014). Differentially expressed genes (DEGs) with |log2FC|≧2 (FC, fold change between groups) and FDR ≤0.05 (FDR, false discovery rate) were considered to be significantly different expressed genes. GO functional analysis and KEGG pathway analysis were then performed for differentially expressed genes. The *p*-values were adjusted using FDR correction, with a threshold of <0.05 considered statistically significant. The DEGs interaction of the growth genes was investigated using the STRING database and Cytoscape v3.10.2 software. The deduced amino acid sequence was submitted to search in the STRING database (www.string-db.org). A PPI network is defined as a graph, where nodes and edges represent genes and their interactions, respectively.

### Experimental validation with qRT-PCR

To verify the reliability of the transcriptome sequencing data. We randomly selected 10 genes with significant differences from the major regulatory pathways associated with brain and muscle growth traits for validation. Primer 5.0 Plus was used to design specific primers for these genes (Supplementary Table S1), and β-actin was used as an internal reference gene. Total RNA was extracted from the samples using a TRIzol Kit. Total RNA was used for cDNA synthesis with PrimeScript™ RT Reagent Kit with gDNA Eraser (Takara, Japan) based on the manufacturer’s instructions. PCR was performed using TB Green® Premix Ex Taq™ II (TaKaRa, Japan) according to the manufacturer’s instructions. The reaction was conducted in a 10 μL volume using a QIAquant 96 2plex Real-time PCR System. The qRT-PCR program was as follows, 95°C for 30 s, followed by 40 cycles of 95°C for 10 s, 60°C for 20 s, and 72°C for 20 s. Each candidate gene was assessed based on three biological replicates. The 2^−ΔΔCT^ method was used to calculate the relative gene expression values. The results of the qRT-PCR analysis are expressed as the means ± SD.

## Result

### HE slices observation

**FIGURE 1:**
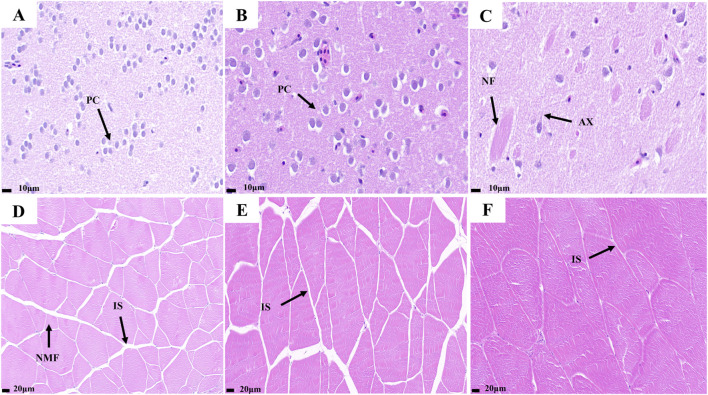
Telencephalon and muscle transverse slices of largemouth bass. **(A)** small fish telencephalon slice; **(B)** medium fish telencephalon slice; **(C)** big fish telencephalon slice; **(D)** small fish muscle transverse slice; **(E)** medium fish muscle transverse slice; **(F)** big fish muscle transverse slice. (PC) Pyramid Cell; (AX) Axon; (NF) Nerve Fibers; (IS) Interosseous space; (NMF) new muscle fiber.

HE staining results of the telencephalon and dorsal muscles of the largemouth bass at the big, medium, and small stages are shown in Figure 1. The density of pyramidal cells in the telencephalons of small fish was greater than that of medium and large fish. In the telencephalon of the big largemouth bass, axons, and nerve fibers were more pronounced than in the other two groups. The results showed that the nervous system in the brain of the fish became more refined and responded more quickly to external stimuli with growth (Figures 1A–C). The results showed that the small largemouth bass presented larger gaps between muscle fibers compared with the large fish and showed many newly generated muscle fibers. During fish growth, the muscle gap continues to narrow and muscles become firmer (Figures 1D–F).

### Preliminary analysis of the transcriptome sequences

Eighteen cDNA libraries were constructed from the total RNA extracted from the brain and muscles of the largemouth bass (Supplementary Table S2). In this experiment, a total of 140.24 Gb of clean data was obtained, and the clean data of each sample reached more than 6.92 Gb. The Q20 values were greater than 98.08%, and the Q30 values were greater than 94.20%, indicating that the sequencing quality was very high. Clean reads were mapped to the reference genome, and the total mapped reads ranged from 91.48% to 96.84%.

### Differential expression analysis

**FIGURE 2:**
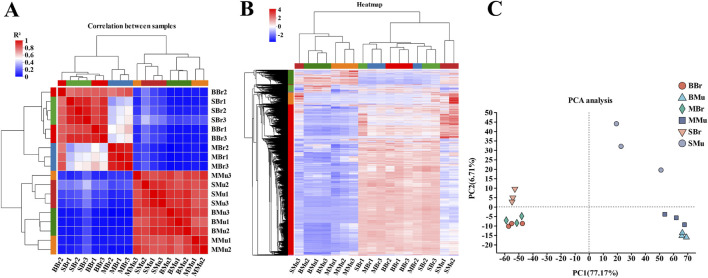
Evaluation of sample transcriptomic correlation. BBr, big largemouth bass’s brain; MBr, medium largemouth bass’s brain; SBr, small largemouth bass’s brain; BMu, big largemouth bass’s muscle; MMu, medium largemouth bass’s muscle; SMu, small largemouth bass’s muscle. **(A)** Pearson correlation coefficients for comparisons among all samples; **(B)** The sample relationship cluster analysis; **(C)** The PCA distribution of 18 samples. In the heat map, each row represents one gene, and each column represents one sample. Different color areas represent different clustering information, and the color from red to blue represents the expression intensity of differentially expressed genes from high to low.

To test the reliability of the samples and determine whether the sample selection was reasonable, a correlation analysis was performed on 18 libraries. The results revealed that the correlation coefficients (*R* ^2^) of the intragroup were all beyond 0.82 (Figure 2A), and the hierarchical clustering analysis of gene expression revealed that the expression patterns were similar within the groups (Figure 2B), indicating that our experimental data were reliable. Furthermore, the PCA analysis also indicated that the replicates of each group had highly repeatable expression (Figure 2C), which indicated that the sample selection and experimentation were reasonable.

**FIGURE 3:**
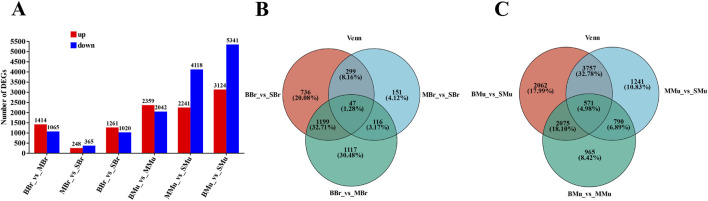
Identification of differentially expressed genes (DEGs) in different groups. **(A)** the pairwise comparison of the numbers of DEGs in different periods of tissues. **(B)** Venn diagram showing the overlapping number of DEGs in the pairwise comparisons. **(C)** Venn diagram showing the overlapping number of DEGs in the pairwise comparisons. All DEGs were determined based on statistical significance according to an FDR<0.05.

We identified 74,781 transcripts, including 50,365 known and 24,416 new transcripts. Pairwise comparisons were then performed of the three stages of largemouth bass brain and muscle tissues (Figure 3A). In the brain tissue, 2,479 DEGs were identified in the BBr (Big fish Brain) vs. MBr (Medium fish Brain) group, of which 1,414 were upregulated and 1,065 were downregulated. 613 DEGs were identified in the MBr vs. SBr (Small fish Brain) group, of which 248 were upregulated and 365 were downregulated. 2,281 DEGs were identified in the BBr vs. SBr group, of which 1,261 were upregulated and 1,020 were downregulated. The results showed that in the muscle tissue, 4,401 DEGs were identified in the BMu (Big fish Muscle) vs. MMu (Medium fish Muscle) group, of which 2,359 were upregulated and 2,042 were downregulated. 6,359 DEGs were identified in the MMu vs. SMu (Small fish Muscle) group, of which 2,241 were upregulated and 4,118 were downregulated. 8,465 DEGs were identified in the BMu vs. SMu group, of which 3,124 were upregulated and 5,341 were downregulated. In addition, we plotted a Venn diagram of the DEGs of these different growth periods in different tissues (Figures 3B,C).

## GO enrichment analysis of DEGs

**FIGURE 4:**
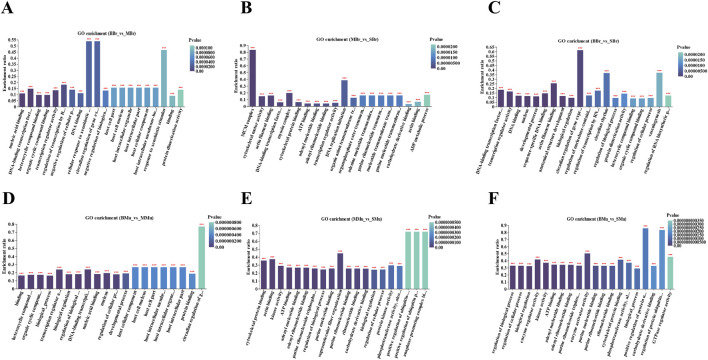
Top 20 terms in the GO term enrichment of significant DEGs in brain and muscle. **(A)** BBr-vs-MBr; **(B)** MBr-vs-SBr; **(C)** BBr-vs-SBr; **(D)** Mu-vs-MMu; **(E)** MMu-vs-SMu; **(F)** BMu-vs-SMu; *X*-axis indicates GO terms while the *Y*-axis indicates the rich factor. Rich factor refers to the ratio of DEGs relative to all genes annotated in the GO term. The higher the rich factor, the greater the intensity. The input number represents the number of DEGs enriched in the specific GO term. *p*-value indicates the enrichment significance of the GO term, and a lower *p*-value represents greater intensity.

We performed GO enrichment analysis of the DEGs in the brain and muscle of largemouth bass. The top 20 GO terms for each comparison are shown in Figure 4. In the brain tissue, nucleic acid binding, DNA-binding transcription factor activity, heterocyclic compound binding, and organic cyclic compound binding were the top four terms in the BBr vs. MBr group (Figure 4A). MCM complex, cytoskeletal motor activity, actin filament binding, and DNA-binding transcription factor activity were the top four terms in the MBr vs. SBr group (Figure 4B). DNA-binding transcription factor activity, transcription regulator activity, DNA binding, and nucleus were the top four terms in the BBr vs. SBr group (Figure 4C). The results showed that in the muscle tissue, binding, heterocyclic compound binding, organic cyclic compound binding, and biological processes were the top four terms in the BMu vs. MMu group (Figure 4D). Cytoskeletal protein binding, actin binding, kinase activity, and ATP binding were the top four terms in the MMu vs. SMu group (Figure 4E). Regulation of biological processes, regulation of cellular processes, biological regulation, and enzyme regulator activity were the top four terms in the BMu vs. SMu group (Figure 4F).

### KEGG enrichment analysis of DEGs

**FIGURE 5:**
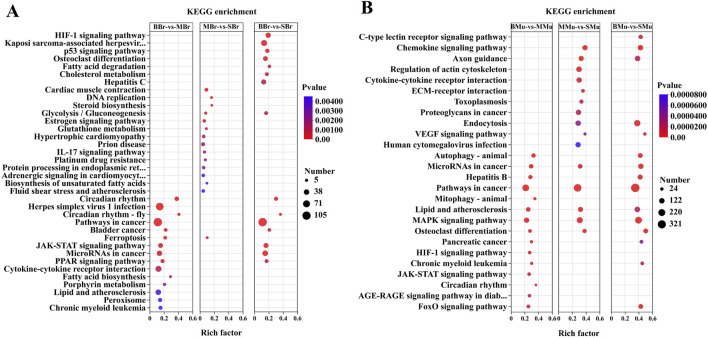
Top 15 pathways in the KEGG enrichment of significant DEGs in brain and muscle. **(A)** KEGG enrichment is the pairwise comparison of the numbers of DEGs in different periods of brain tissues; **(B)** KEGG enrichment is the pairwise comparison of the numbers of DEGs in different periods of muscle tissues. The *Y*-axis represents the KEGG pathway while the *X*-axis represents the rich factor. Rich factor indicates the ratio of DEGs relative to all genes enriched in the specific pathway. The higher the rich factor, the greater the intensity. The input number represents the number of DEGs enriched in the specific pathway. *p*-value represents the enrichment significance of the signaling pathway, and a lower *p*-value represents greater intensity.

KEGG pathway analysis was performed to further identify the metabolic pathways in largemouth bass, and the top 15 enriched pathways are shown in Figure 5. In the brain tissue (Figure 5A), the top five pathways in the BBr vs. MBr group were circadian rhythm, herpes simplex virus 1 infection, circadian rhythm fly, cancer, and bladder cancer pathways. The top five pathways in the MBr vs. SBr group were cardiac muscle contraction, DNA replication, steroid biosynthesis, glycolysis/gluconeogenesis, and estrogen signaling pathways. The top five pathways in the BBr vs. SBr group were HIF-1 signaling, Kaposi sarcoma-associated herpes virus infection, p53 signaling, osteoclast differentiation, and fatty acid degradation pathways.

In the muscle tissue (Figure 5B), the top five pathways in the BMu vs. MMu group were autophagy-animal, microRNAs in cancer, hepatitis B, cancer, and mitophagy-animal pathways. The top five pathways in the MMu vs. SMu group were chemokine signaling, axon guidance, regulation of the actin cytoskeleton, cytokine-cytokine receptor interaction, and ECM-receptor interaction pathways. The top five pathways in the BMu vs. SMu groups were the C-type lectin receptor signaling, chemokine signaling, axon guidance, endocytosis, and VEGF signaling pathways. A comparison of the three groups showed that the common pathways were microRNAs in cancer, pathways in cancer, lipid, and atherosclerosis, MAPK signaling, and osteoclast differentiation pathways.

### Protein-protein interaction (PPI) analysis and growth gene prediction

**FIGURE 6:**
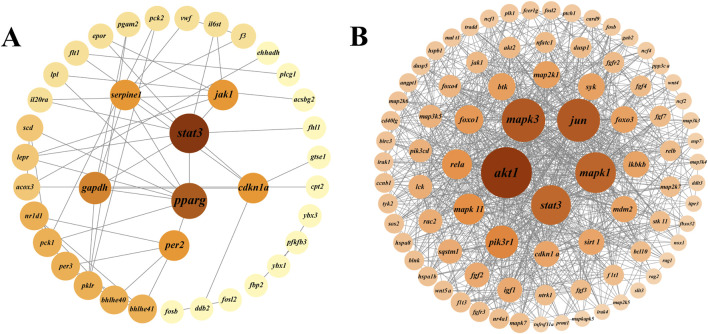
PPI networks of DEGs in brain **(A)** and muscle **(B)** growth-related pathways.

**FIGURE 7:**
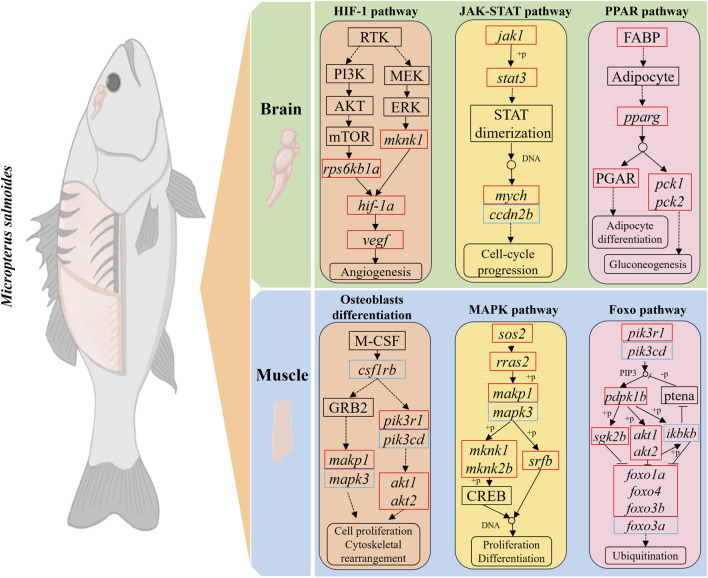
Schematic representation of DEGs in growth-related pathways. Red and blue colors indicate upregulation and downregulation of DEGs, respectively.

Protein-protein interaction (PPI) analysis of DEGs in the growth-related pathways of largemouth bass was performed using the STRING database and Cytoscape software to further explore the interactions between DEGs (Figure 6). In the BBr vs. SBr group, DEGs of eight pathways (HIF-1 signaling, osteoclast differentiation, p53 signaling, glycolysis/gluconeogenesis, circadian rhythm, circadian rhythm-fly, JAK-STAT signaling, and PPAR signaling pathways) were selected to generate the PPI network (Figure 6A). Results were obtained for a total of 38 nodes, and *stat3*, *pparg*, *per2*, *gapdh*, *cdkn1a*, *serpne1*, and *jak1* ranked the highest. In the BMu vs. SMu group, DEGs from five pathways (osteoclast differentiation, MAPK signaling, FoxO signaling, C-type lectin receptor signaling, and axon guidance pathways) were selected to generate the PPI network (Figure 6B). The results showed that *akt1*, *mapk3*, *mapk1*, *stat3*, and *jun* ranked the highest. To visualize cell proliferation and differentiation more clearly, we mapped the relevant pathways based on the expression of DEGs (Figure 7).

### Validation by real-time PCR

**FIGURE 8:**
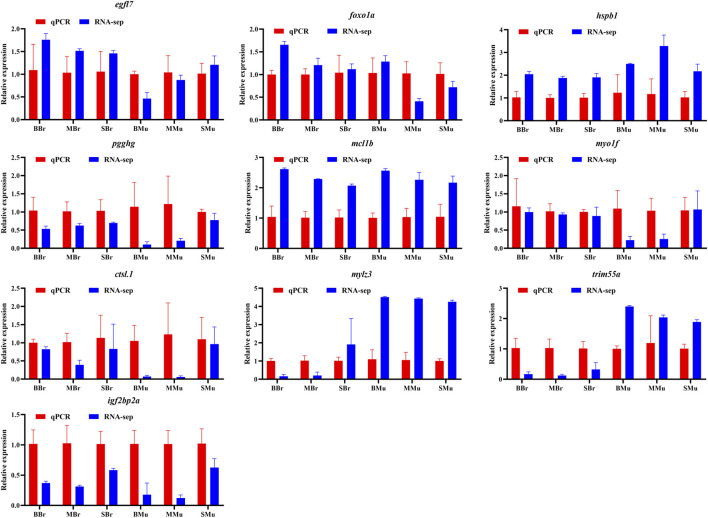
Validation of RNA-seq expression values by qPCR.

To validate the gene expression of the transcriptome data, 10 genes were selected for real-time PCR of the muscle and brain tissue of largemouth bass from three different periods. The expression patterns of these genes detected by qRT-PCR were strongly correlated with the transcriptome sequencing data (Figure 8). Thus, the qRT-PCR results were consistent with the transcriptome analysis, thereby supporting the reliability of the transcriptome data.

## Discussion

In this study, the brain and muscle transcriptomes of largemouth bass at different growth stages were comparatively analyzed for the first time using RNA sequencing to reveal the mechanisms underlying growth differences at different developmental stages. The aim of this study was to investigate the expression patterns of growth-related genes and their variability in different tissues, thereby providing insights into the physiological regulatory mechanisms of largemouth bass at different developmental stages.

Growth is an important and complex quantitative trait for economically valuable aquaculture species, and it is influenced by many external and internal factors (Shi et al., 2024; Lu et al., 2020; Yin et al., 2023). Studies have found that certain external factors have significant effects on the growth of largemouth bass. For example, under high-temperature conditions of 30°C, feeding 3% of the total weight of the fish every day or feeding every other day until full can effectively ensure the healthy growth of largemouth bass. The interaction between high temperatures and hypoxia has severe negative effects on the gills and liver of largemouth bass by increasing oxidative stress, suppressing immune function, and increasing apoptosis. In addition, external factors such as nutrition, light, dissolved oxygen, and exercise intensity can affect the growth and development of largemouth bass (Liu et al., 2024; Tuttle et al., 2022; Chen F. et al., 2024; Cao et al., 2024). Intrinsic factors also play key roles in determining the growth potential of fish and their ability to adapt to the external environment. Intrinsic factors include genetic background, endocrine regulation, metabolic efficiency, and physiological health status (Lu et al., 2022).

Hematoxylin and eosin (HE) staining is a commonly used histological technique that provides a wealth of information on cell and tissue morphology through microscopic observations of HE-stained tissue sections (Kim, 2021). HE staining has been widely used in fish histological studies (Han et al., 2024; Li Q. et al., 2024). Muscle growth in fish typically occurs via two pathways: proliferation and hypertrophy. During the juvenile stage, muscle growth mainly occurs through the augmentation of muscle cells (proliferation), whereas after entering the growth stage, muscle growth is dominated by an increase in the diameter of muscle fibers (hypertrophy) (Li et al., 2023; Zhang et al., 2023). In this experiment, the results of muscle HE staining showed significant differences among the small, medium, and big groups of largemouth bass. Many newly generated muscle fibers were observed in the muscles of the small largemouth bass, whereas only muscle hyperplasia was observed in the other two groups. Therefore, we hypothesized that largemouth bass completed myofiber proliferation before 1.5 months of age and muscle growth was subsequently dominated by hypertrophy. The brain HE staining results showed that big largemouth bass have more complex nerve fibers, suggesting that they are more adaptable to complex environments. In *Colossoma macropomum* study, an increase in the number of telencephalic cells was found to coincide with enhanced spatial learning and memory, in agreement with the results of this experiment (Pereira et al., 2020). However, the specific mechanism underlying this developmental process is still not fully understood, thus, more in-depth studies and analyses are needed to reveal the underlying molecular mechanisms and regulatory pathways.

The brain is the central organ of the vertebrate central nervous system. In fish, the brain controls aspects of their growth, reproduction, metabolism, locomotion, and cognitive behavior by regulating endocrine signals and neurotransmitters (Colin-Garcia et al., 2023; Oka, 2023; Lucon-Xiccato et al., 2023). In zebrafish, newborn neurons have multiple functions, including learning, memory, and sensory processing, and some adult teleosts fish present significant neurogenesis brain (Kizil et al., 2012). The KEGG enrichment analysis showed that DEGs in the brains of small and big largemouth bass were significantly enriched in the HIF-1, p53, JAK-ATAT, and PPAR signaling pathways, thus, these pathways are likely related to fish brain development.

HIF-1α, as a major response factor to hypoxia, regulates a series of genes related to cell proliferation, metabolism, and survival. Upregulation of HIF-1α and activation of the Wnt/β-catenin pathway together promote neural stem cell proliferation (Qi et al., 2017). PPAR activation significantly increases the proliferation rate of neural stem cells and promotes the generation of neurons during the differentiation of neural stem cells, whereas it inhibits the differentiation of glial cells (Qi et al., 2017). These pathways are all involved in the development of the fish nervous systems and indirectly affect memory, learning, and behavior by controlling synapses and neurons. Combining the results of previous studies with ours, we speculate that neural development may be the key to the growth of largemouth bass. Signal transduction and activator of transcription (Dillies et al., 2013) factors have important effects on a variety of physiological processes, such as nervous system development, immune response, and cell proliferation and differentiation. Stat3 is a member of the *stat* family, and activated *stat3* can promote axonal growth and neuronal survival (Fukuda et al., 2007; Foshay, 2008). In this study, we observed a gradual increase in the expression of *stat3* from the brains of largemouth bass at the three different growth stages. Thus, neurodevelopment plays an important role in bass growth.

The brain is one of the most expensive organs in terms of energy expenditure in vertebrates (Belanger et al., 2011). Neuronal membrane potential maintenance in the brain and synaptic transmissions are accompanied by energy expenditure. The gene *pparg* is a key regulator of lipid metabolism and modulates the expression of a variety of genes related to fatty acid metabolism. By activating these genes, *pparg* promotes the metabolic utilization of fatty acids and ensures that the brain receives sufficient energy (Belanger et al., 2011). The gene *gapdh* is critical for the glycolytic pathway, which converts glucose into pyruvate and generates ATP as an energy source. In the brain, where energy demand is high, the role of *gapdh* in glycolysis is essential for maintaining cellular energy levels. The main role of *egfl7* is to promote angiogenesis, and when *egfl7* gene expression is suppressed, microvessel production in the brain is significantly affected. The vascular network provides essential oxygen and nutrients to the brain tissue and carries metabolic waste away from it (Hao et al., 2024). In the present study, the brain of small largemouth bass showed enhanced lipid metabolism and decreased glucose metabolism compared with that of big largemouth bass. Therefore, we hypothesized that the brain of juvenile largemouth bass obtains energy from carbohydrate metabolism through blood vessels. With growth, the vasculature increases and requires more energy, which is not sufficiently met by carbohydrate metabolism, therefore, lipid metabolism may become the main source of energy for the fish brain. The combined results suggest that the brain continues to build complex neural and vascular networks during largemouth bass growth to support the increasingly complex behavioral and cognitive functions of fish.

Fish muscles account for the vast majority of their body weight, therefore, fish growth is largely reflected in the growth of muscle tissue (Yao et al., 2025; Shakur Ahammad et al., 2019). Muscle growth involves complex physiological and biochemical pathways. In fish, the MAPK signaling pathway has been shown to have an important regulatory role in muscle growth, organ development, and regeneration. In particular, the MAPK pathway promotes muscle fiber formation and growth by regulating the expression of specific genes during the proliferation and differentiation of fish muscle cells (Sun et al., 2014). In our study, the MAPK, osteoclast differentiation, and FoxO signaling pathways were similarly associated with the growth of largemouth bass.

We further analyzed the DEGs in largemouth bass growth-related pathways. The results showed that *akt1*, *mapk3*, *mapk1*, and *jun* were significantly associated with the growth of largemouth bass. *Akt1* (protein kinase B1) is a key cellular signaling molecule, and the PI3K/AKT/mTOR signaling pathway promotes cell growth and proliferation. Studies on zebrafish showed that *akt1* synergizes with the mTOR pathway to promote protein synthesis and muscle growth (Cheng et al., 2013; Jung et al., 2013). A study on hybrid catfish revealed that the addition of leucine to the diet increased muscle protein synthesis by regulating the AKT/TOR signaling pathway and attenuated protein degradation by regulating the AKT/FOXO3a signaling pathway (Zhao et al., 2020). In our experiments, the expression level of *akt1* in the muscle tissue gradually increased with largemouth bass growth, while that of *foxo3a* showed a decreasing trend. *Mapk3* and *mapk1* are important members of the MAPK signaling pathway and promote the proliferation of myogenic precursor cells by activating the ERK/MAPK signaling pathway (Zhao H. et al., 2024; Fang et al., 2023). Myogenic factor 6 (*myf6*) is a muscle-specific transcription factor that belongs to the myotonin family. MAPK3/MAPK1 regulates the activity and expression of *myf6* through the ERK pathway, thereby affecting myoblast-specific gene expression to promote the differentiation of myoblasts into mature myofibroblasts (Yao et al., 2025; Li Q. et al., 2024). In this study, the expression of *myf6* in the muscle gradually increased with the growth of largemouth bass. The combined results showed that the rate of protein synthesis increased in largemouth bass and that the muscles became more mature and better able to adapt to external loads and stressful conditions.

## Conclusion

During the early developmental stages of largemouth bass before 1.5 months of age, muscle growth is dominated by myoblast hyperplasia, whereas during the later growth stages, muscle growth relies primarily on myofiber hypertrophy. The nervous system in the brain of largemouth bass is more developed in big fish. Comparative RNA-seq analysis identified 19,225 and 5,373 DEGs in the muscle and brain tissue, respectively. We found that certain crucial pathways were associated with growth and myogenesis, including MAPK, osteoclast differentiation, glycolysis/gluconeogenesis, JAK-ATAT signaling, and PPAR signaling pathways. PPI analysis revealed that *stat3*, *pparg*, *akt1*, *mapk3*, and *mapk1* were among the key hub genes. The present study provides useful transcriptomic resources for further research on the molecular mechanisms of largemouth bass growth and genetic improvement of largemouth bass.

## Data Availability

The datasets presented in this study can be found in online repositories. The names of the repository/repositories and accession number(s) can be found below: https://www.ncbi.nlm.nih.gov/, PRJNA1148318.
