# Supplementary material for: Identification of key genes related to growth of largemouth bass (Micropterus salmoides) based on comprehensive transcriptome analysis
Source: Front Mol Biosci. 2024 Dec 11;11:1499220. doi: 10.3389/fmolb.2024.1499220 (PMC11670207; doi:10.3389/fmolb.2024.1499220)
Supplement: Supplementary file 1 [file Table1.docx]

**Supplementary Table S1**

Primer Sequences for quantitative RT-PCR validation.

| genes | Forward primers  (5′→ 3′) | Reverse primers  (5 → 3′) | Product size/bp | Annealing temperature (◦C) |
| --- | --- | --- | --- | --- |
| *egfl7* | AACTGCAACCAAGCTGTGTG | TCGCTACACTCATCCACATCTG | 128 | 60 |
| *foxo1a* | ACAGCAAGTTCACCAAGAGC | ACGCATCAAAGTCGTCGTTG | 147 | 60 |
| *hspb1* | AGCGGCATGTCAGAAATCAAG | TTTCCAACACACCGTCCTTG | 112 | 60 |
| *pgghg* | AAGCTGGTCAGTTCGTTCAC | TGGAAGCAGTGTCAAAGTGG | 177 | 58 |
| *mcl1b* | CCGCGGAAAGAAAACAAAGC | ACAAAACTGGCATCGTCTCC | 134 | 60 |
| *myo1f* | AGACAGTGGCTGCCAAATAC | TTGGCATTCCCGAAAGCTTC | 127 | 59 |
| *ctsl.1* | GTGCCTTCCAGTACATTCAAGC | TGCAAATGGCTCCAATGGTG | 114 | 60 |
| *mylz3* | TTGACAGAGTTGGTGACAGC | AGGAAAGCGTCGAAGTTGAG | 157 | 59 |
| *trim55a* | AAACACAGAAGGCAGAGCTG | TTCTGTCTGCGTCCGTTTTC | 127 | 59 |
| *igf2bp2a* | AAATCCTCGCTCACAACAGC | GCTTCGGCTTTACAACATGC | 184 | 59 |
| *β-actin* | AACACTGTGCTGTCTGGAGG | CGGACTCATCGTACTCCTGC | 210 | 60 |
