# Supplementary material for: Identification of key genes related to growth of largemouth bass (Micropterus salmoides) based on comprehensive transcriptome analysis
Source: Front Mol Biosci. 2024 Dec 11;11:1499220. doi: 10.3389/fmolb.2024.1499220 (PMC11670207; doi:10.3389/fmolb.2024.1499220)
Supplement: Supplementary file 2 [file Table2.docx]

**Supplementary Table S2**

Summary of transcriptome data generated from largemouth bass samples.

| Sample | Raw reads | Clean reads | Q20(%) | Q30(%) | GC content(%) | Total mapped |
| --- | --- | --- | --- | --- | --- | --- |
| BBr1 | 46610402 | 46180012 | 98.46 | 95.18 | 46.82 | 44251780(95.82%) |
| BBr2 | 56359568 | 55879564 | 98.38 | 95.01 | 47.45 | 52831731(94.55%) |
| BBr3 | 49027840 | 48601804 | 98.08 | 94.20 | 47.06 | 46706240(96.10%) |
| MBr1 | 52859152 | 52269764 | 98.15 | 94.42 | 47.93 | 49314390(94.35%) |
| MBr2 | 51495388 | 51075830 | 98.39 | 95.02 | 47.85 | 49201311(96.33%) |
| MBr3 | 56483514 | 56027342 | 98.28 | 94.72 | 47.51 | 53821805(96.06%) |
| SBr1 | 48395370 | 47806504 | 98.16 | 94.43 | 46.38 | 45877029(95.96%) |
| SBr2 | 53488834 | 52901046 | 98.12 | 94.36 | 47.72 | 50807900(96.04%) |
| SBr3 | 54082366 | 53673144 | 98.42 | 95.07 | 47.87 | 51502708(95.96%) |
| BMu1 | 49758452 | 49356244 | 98.61 | 95.61 | 51.32 | 47657804(96.56%) |
| BMu2 | 46605952 | 46152242 | 98.31 | 94.84 | 51.54 | 44559256(96.55%) |
| BMu3 | 50603764 | 50166492 | 98.40 | 95.08 | 51.23 | 48458265(96.59%) |
| MMu1 | 53023696 | 52518140 | 98.57 | 95.48 | 52.1 | 50859386(96.84%) |
| MMu2 | 52062602 | 51635118 | 98.58 | 95.46 | 51.67 | 49848664(96.54%) |
| MMu3 | 60132454 | 59501846 | 98.51 | 95.35 | 51.97 | 54434279(91.48%) |
| SMu1 | 54746714 | 54289870 | 98.54 | 95.40 | 50.9 | 52504000(96.71%) |
| SMu2 | 55981884 | 55523500 | 98.53 | 95.36 | 50.49 | 53634011(96.60%) |
| SMu3 | 54529054 | 53746672 | 98.48 | 95.38 | 51.58 | 50634454(94.21%) |
